# Supplementary material for: An antioxidant screen identifies ascorbic acid for prevention of light-induced mitotic prolongation in live cell imaging
Source: Commun Biol. 2023 Nov 1;6:1107. doi: 10.1038/s42003-023-05479-6 (PMC10620154; doi:10.1038/s42003-023-05479-6)
Supplement: Supplementary file 2 — Supplementary Information [file 42003_2023_5479_MOESM2_ESM.pdf]

Supplementary Information

An antioxidant screen identifies ascorbic acid for prevention of light-induced mitotic prolongation in live cell imaging

Tomoki Harada[1,#], Shoji Hata[1,2,#,*], Rioka Takagi[1], Takuma Komori[1], Masamitsu Fukuyama[1], Takumi Chinen[1], Daiju Kitagawa[1,*]

[1]Department of Physiological Chemistry, Graduate School of Pharmaceutical Sciences, The University of Tokyo, Bunkyo, Tokyo, Japan.

[2]Precursory Research for Embryonic Science and Technology (PRESTO) Program, Japan Science and Technology Agency, Honcho Kawaguchi, Saitama, Japan

[#]Contributed equally

*Correspondence: s.hata@mol.f.u-tokyo.ac.jp

: dkitagawa@mol.f.u-tokyo.ac.jp

ORCID ID of S. Hata: orcid.org/0000-0003-4094-0489

ORCID ID of D. Kitagawa: orcid.org/0000-0003-2509-5977

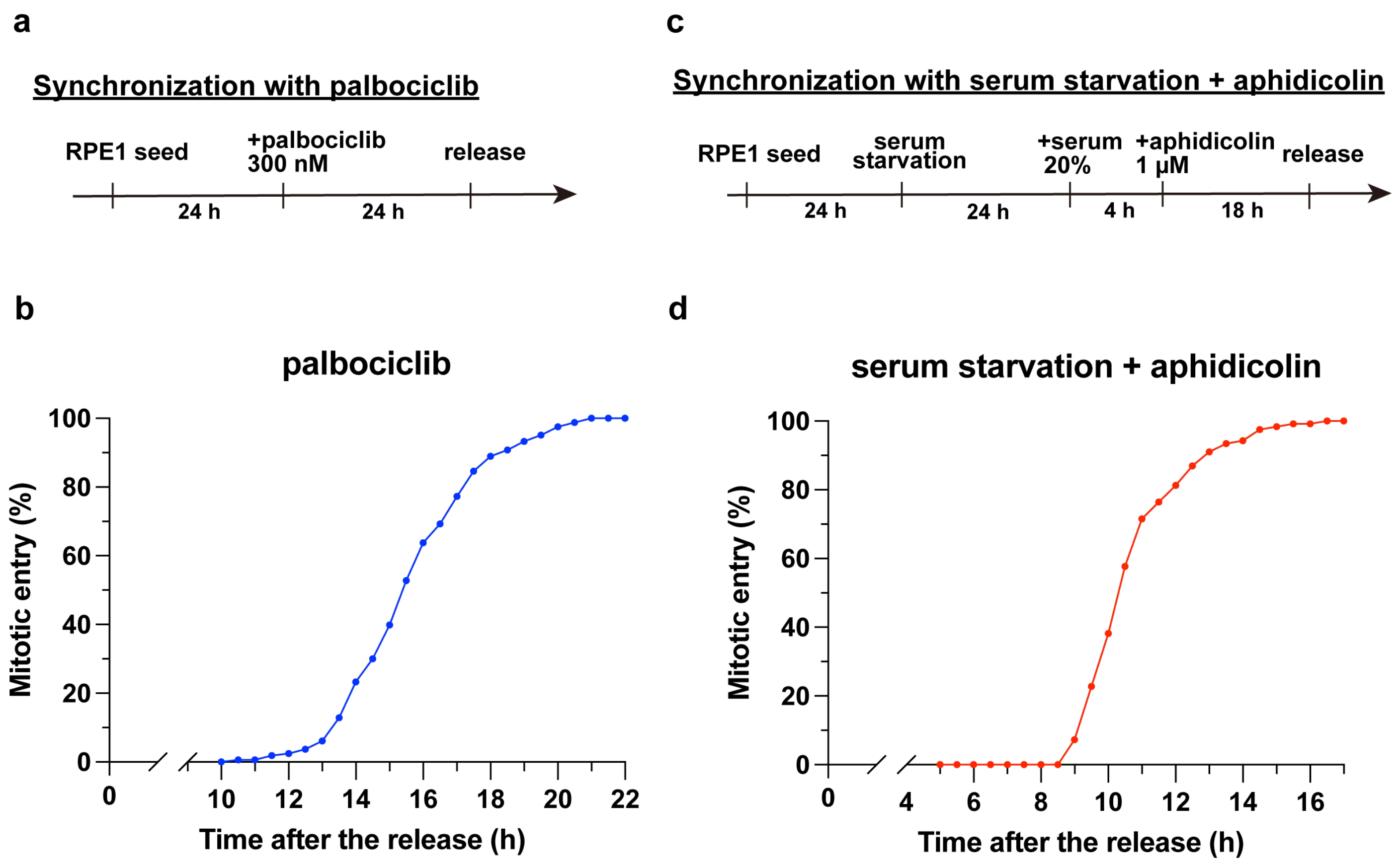

Supplementary Fig. 1: Timing of mitotic entry of the cells synchronized with palbociclib or serum starvation plus aphidicolin.

a Experimental procedure for cell-cycle synchronization with palbociclib treatment. **b** The percentage of cells that entered mitosis after the release from G1 arrest with palbociclib. Live cell imaging was started 10 h after the palbociclib washout. **c** Experimental procedure for cell-cycle synchronization with serum starvation plus aphidicolin treatment. **d** The percentage of cells that entered mitosis after the release from G1/S arrest with serum starvation and aphidicolin. Live cell imaging was started 5 h after the aphidicolin washout.

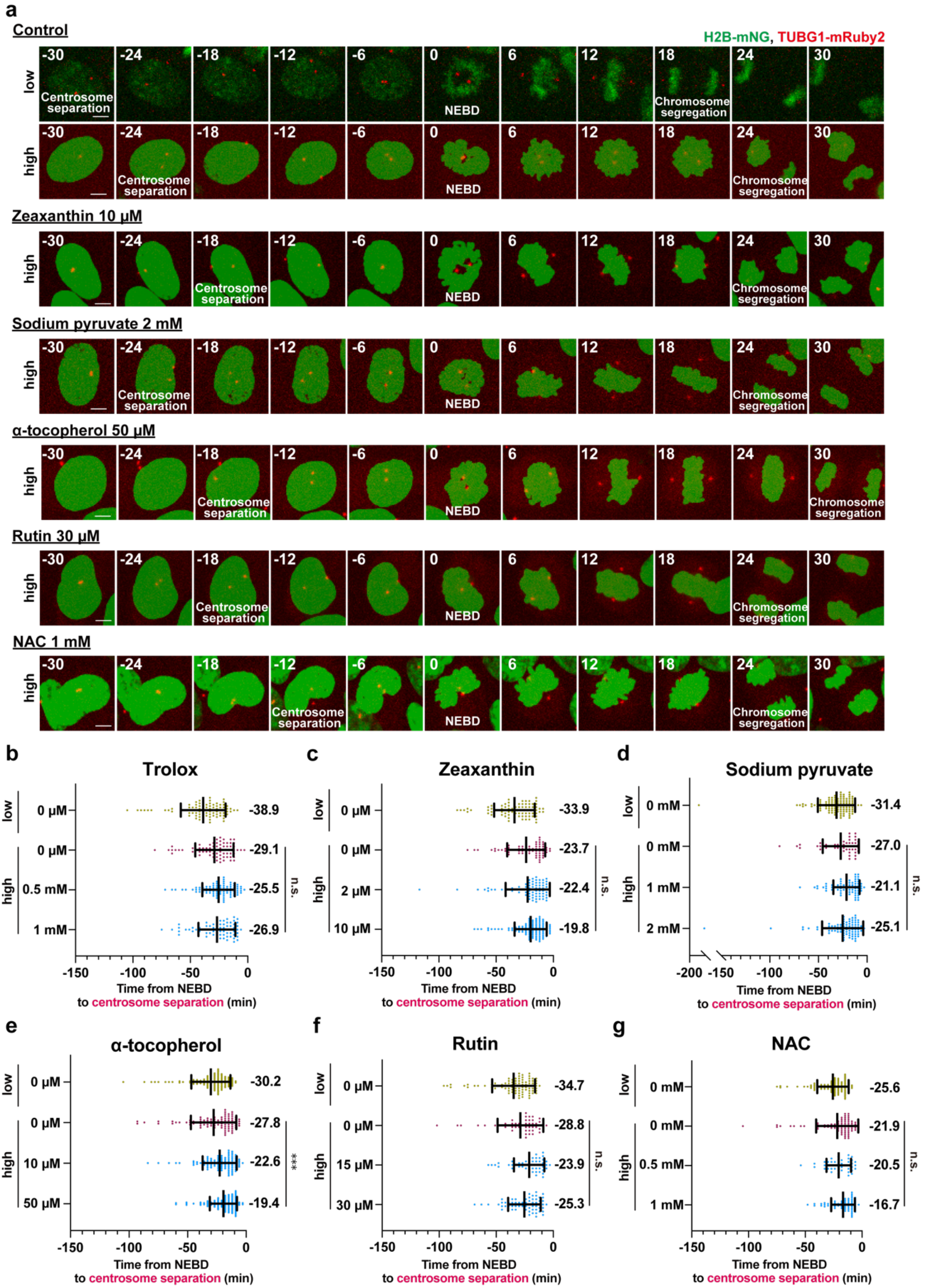
a
Control
H2B-mNG, TUBG1-mRuby2
low
high
-30
-24
-18
-12
-6
0
6
12
18
24
30
Centrosome separation
NEBD
Chromosome segregation
Zeaxanthin 10 µM
Sodium pyruvate 2 mM
α-tocopherol 50 µM
Rutin 30 µM
NAC 1 mM
b
Trolox
0 µM
0.5 mM
1 mM
-38.9
-29.1
-25.5
-26.9
n.s.
c
Zeaxanthin
0 µM
2 µM
10 µM
-33.9
-23.7
-22.4
-19.8
n.s.
d
Sodium pyruvate
0 mM
1 mM
2 mM
-31.4
-27.0
-21.1
-25.1
n.s.
e
α-tocopherol
0 µM
10 µM
50 µM
-30.2
-27.8
-22.6
-19.4
***
f
Rutin
0 µM
15 µM
30 µM
-34.7
-28.8
-23.9
-25.3
n.s.
g
NAC
0 mM
0.5 mM
1 mM
-25.6
-21.9
-20.5
-16.7
n.s.
-200
-150
-100
-50
0
Time from NEBD
to centrosome separation (min)

Supplementary Fig. 2: Antioxidants except for ascorbic acid do not suppress the delay of centrosome separation induced by high-light illumination.

a Time-lapse imaging of mitotic cells in the presence of the indicated antioxidants. Representative still images of cells for each condition are shown (3-min intervals). The brightness of the green channel was reduced to better visualize the red channel. Images with different settings of brightness and contrast are shown. T=0 is designated as NEBD (time shown in min). Scale bar, 5 μm. **b-g** Quantification of mitotic durations in the presence of the following antioxidants from **a**. **b**: Trolox, **c**: Zeaxanthin, **d**: Sodium pyruvate, **e**: α-tocopherol, **f**: Rutin and **g**: NAC. n>40 cells from three independent experiments or more. In **b-g**, data are mean ± S.D., and *P* values were calculated by Mann-Whitney *U*-test. ***P < 0.001, n.s.: not significant.

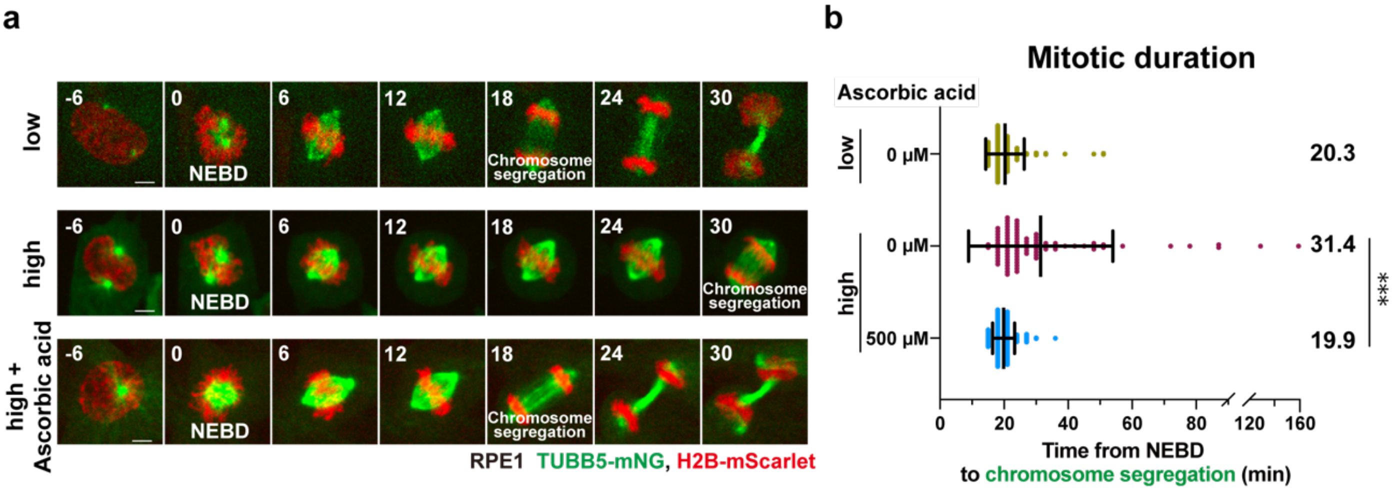

c

BJ-5ta cells

Light condition	Excitation	Laser power	Exposure time	Z-stack	Intervals
low	488 nm	15% (0.70 W/cm²)	50 msec	1 μm step × 21 slices	3 min
	561 nm	20% (0.91 W/cm²)	100 msec		
high	488 nm	100% (4.67 W/cm²)	150 msec	1 μm step × 21 slices	3 min
	561 nm	20% (0.91 W/cm²)	100 msec		

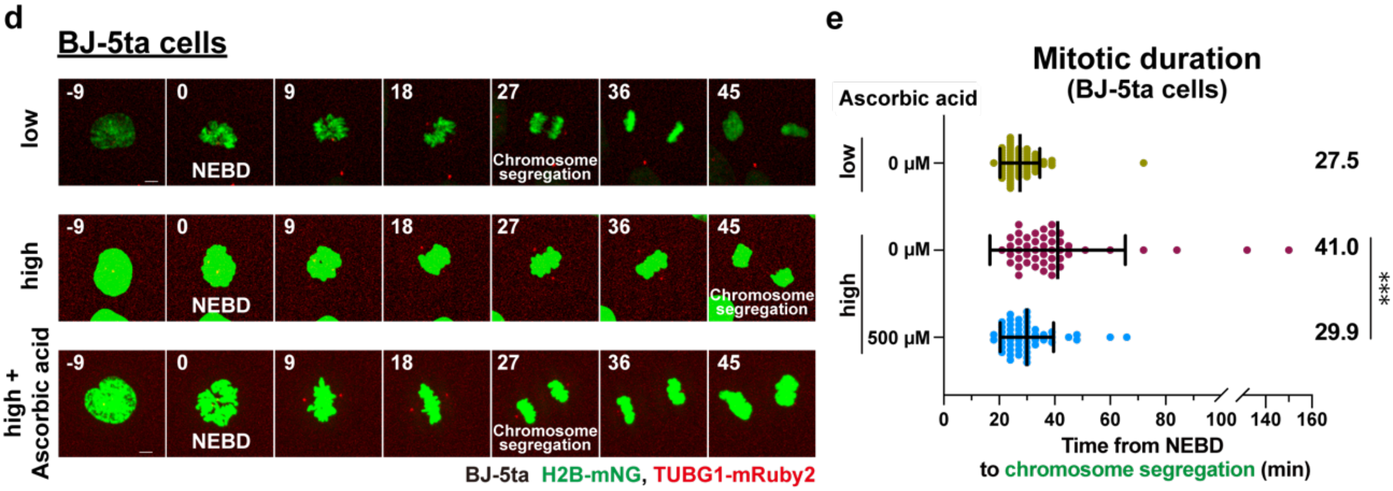

Supplementary Fig. 3: Universal suppressive effect of ascorbic acid on light-induced mitotic prolongation.

a Time-lapse imaging of mitotic RPE1 cells stably expressing TUBB5-mNG and H2B-mScarlet in the low and high conditions (3-min intervals). Representative still images with different settings of brightness and contrast are shown. T=0 is designated as NEBD (time shown in min). Scale bar, 5 μm. **b** Quantification of mitotic duration from **a**. The time from NEBD to chromosome segregation was measured. n>80 cells from three independent experiments. **c** The imaging condition used for live cell imaging of BJ-5ta cells. **d** Time-lapse imaging of mitotic BJ-5ta cells stably expressing H2B-

mNG and TUBG1-mRuby2 in the low and high conditions (3-min intervals). Representative still images with different settings of brightness and contrast are shown. T=0 is designated as NEBD (time shown in min). Scale bar, 5 µm. **e** Quantification of mitotic duration from **d**. The time from NEBD to chromosome segregation was measured. n>45 cells from three independent experiments. In **b** and **e**, data are mean ± S.D., and *P* values were calculated by Mann-Whitney *U*-test. ***P < 0.001, n.s.: not significant.

a

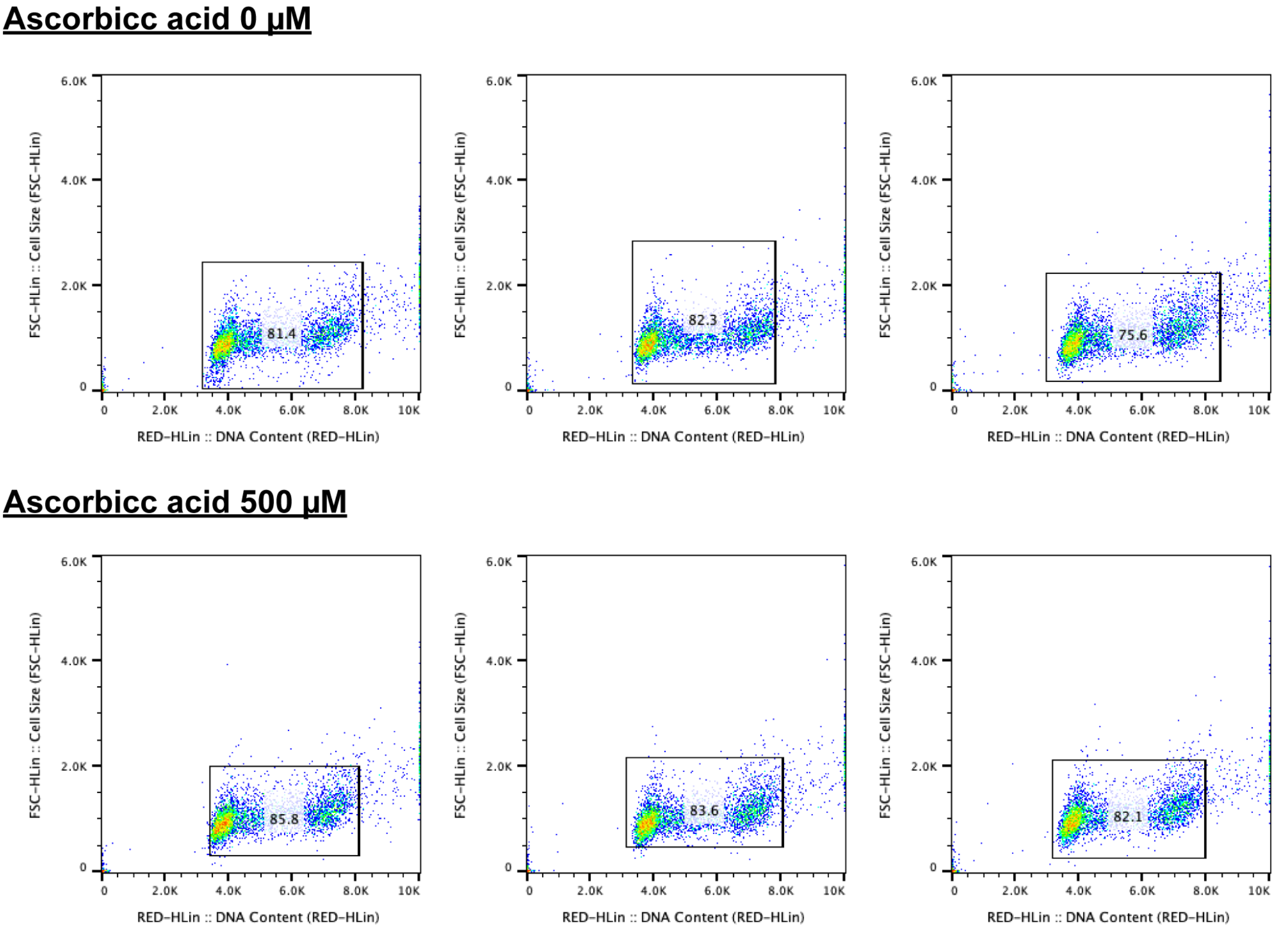

Supplementary Fig. 4: Gating strategy of flow cytometry.

a Populations of RPE1 cells with and without ascorbic acid. Gates and regions were placed around populations of cells with appropriate size and DNA contents, according to the protocol of Muse Cell Cycle Assay.

a Short-interval condition

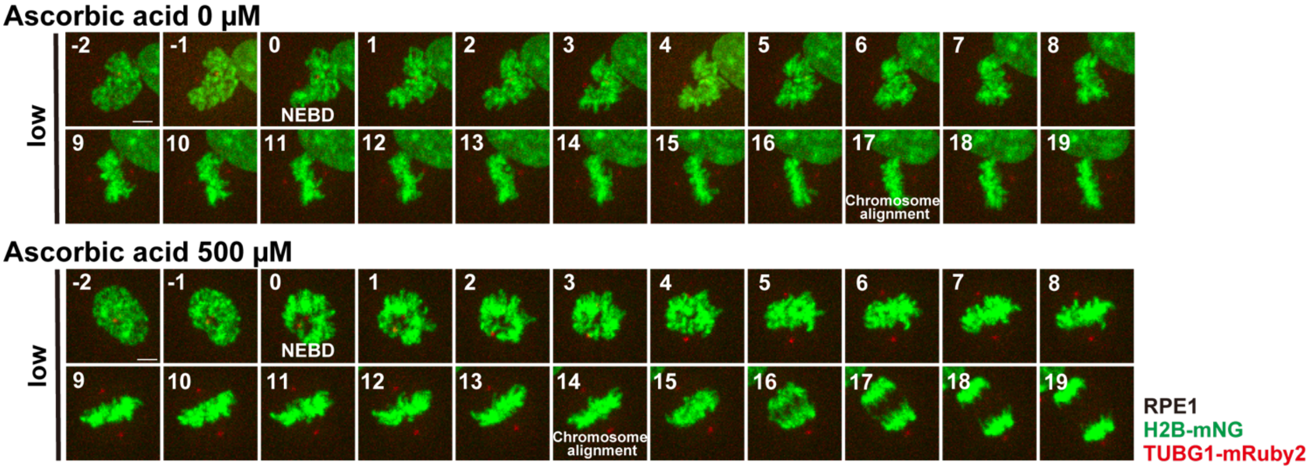

b

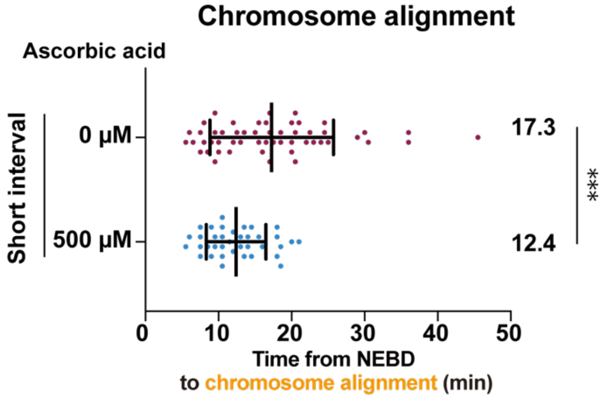

c

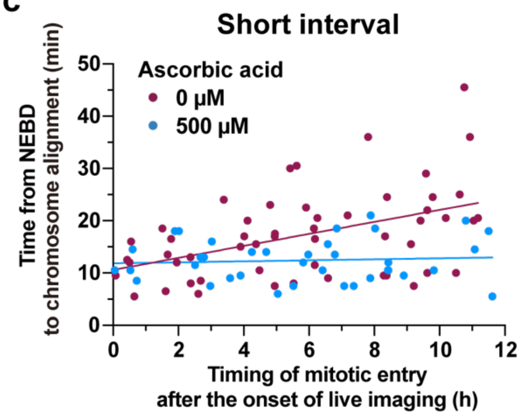

d Short-interval condition for RPE1 TUBB5-mNG H2B-mScarlet

Light condition	Excitation	Laser power	Exposure time	Z-stack	Intervals
low	488 nm	20% (0.93 W/cm²)	50 msec	1 μm step × 21 slices	30 sec
	561 nm	25% (1.14 W/cm²)	75 msec		

e

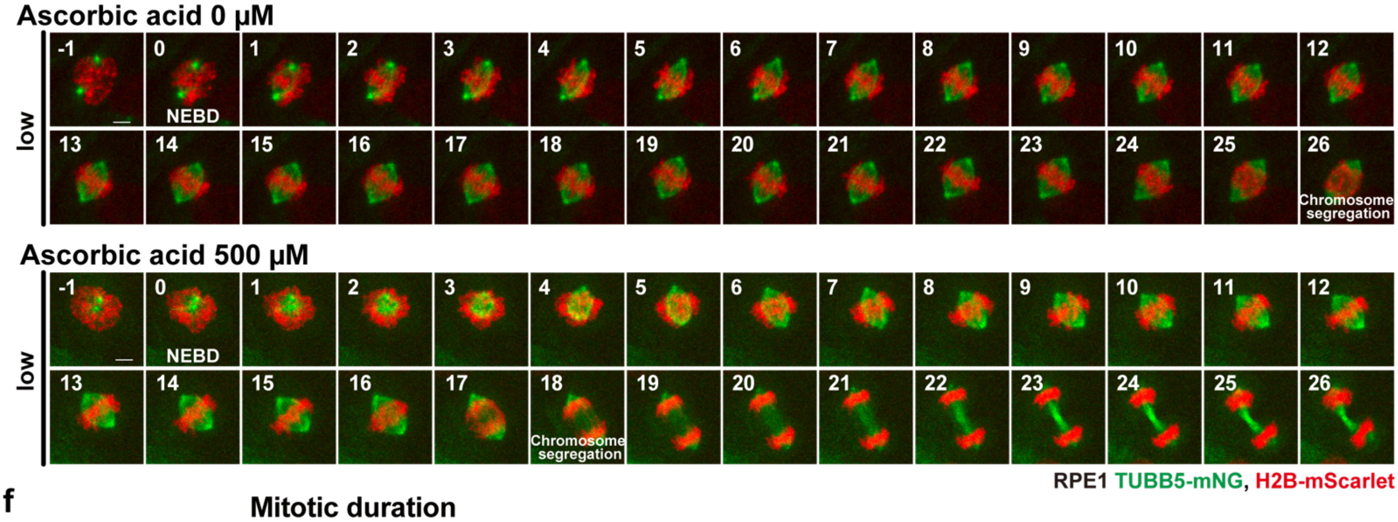

f

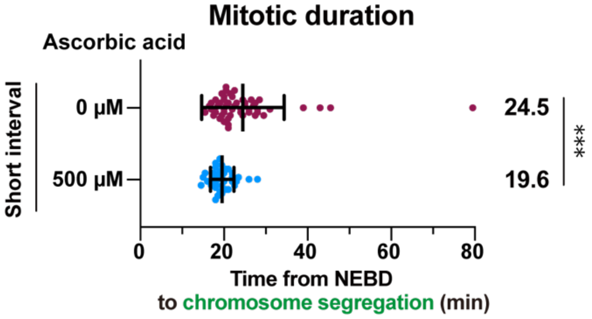

Supplementary Fig. 5: Ascorbic acid alleviates mitotic abnormalities caused by short-interval live imaging.

a Time-lapse images from NEBD to chromosome alignment in the short-interval condition (30-sec intervals). Representative still images with different settings of brightness and contrast are shown. T=0 is designated as NEBD (time shown in min). Scale bar, 5 μm. **b** Quantification of the time required for chromosome alignment from **a**. The time from NEBD to chromosome alignment at the metaphase plate was measured. n>30 cells from three independent experiments. **c** The correlation between the timing of mitotic entry (NEBD) after the start of live imaging (x-axis) and the time required for chromosome alignment (y-axis) in the short-interval condition from **a**. The regression lines for the indicated conditions are shown. **d** The imaging condition used for live cell imaging of RPE1 cells stably expressing TUBB5-mNG and H2B-mScarlet with 30-sec intervals. **e** Time-lapse imaging of mitotic RPE1 cells expressing TUBB5-mNG and H2B-mScarlet in the short-interval condition (30-sec intervals). Representative still images with different settings of brightness and contrast are shown. T=0 is designated as NEBD (time shown in min). Scale bar, 5 μm. **f** Quantification of mitotic duration from **e**. The time from NEBD to chromosome segregation was measured. n>35 cells from four independent experiments. In **b** and **f**, data are mean ± S.D., and *P* values were calculated by Mann-Whitney *U*-test. ***P < 0.001.
