# Supplementary material for: An antioxidant screen identifies ascorbic acid for prevention of light-induced mitotic prolongation in live cell imaging
Source: Commun Biol. 2023 Nov 1;6:1107. doi: 10.1038/s42003-023-05479-6 (PMC10620154; doi:10.1038/s42003-023-05479-6)
Supplement: Supplementary file 3 — Description of Additional Supplementary Files [file 42003_2023_5479_MOESM3_ESM.pdf]

Description of Additional Supplementary Files

File name: Supplementary Data 1
Description: The source data behind the graphs in the paper.

File name: Supplementary Movie 1
Description: Mitotic progression of RPE1 cells in the low- and high-light illumination.
Time-lapse imaging of mitotic RPE1 cells expressing H2B-mNG and TUBG1-mRuby2 in the low and high conditions (3-min intervals). T=0 is assigned as NEBD. The movie corresponds to Fig. 1b.

File name: Supplementary Movie 2
Description: Mitotic progression under the cell-cycle synchronization.
Time-lapse imaging of mitotic RPE1 cells expressing H2B-mNG and TUBG1-mRuby2 synchronized with 300 nM palbociclib or with serum starvation plus 1 μM aphidicolin under the high illumination conditions (3-min intervals). T=0 is assigned as NEBD. The movie corresponds to Fig. 2a, f.

File name: Supplementary Movie 3
Description: Effect of antioxidants against light-induced mitotic abnormalities.
Time-lapse imaging of mitotic RPE1 cells expressing H2B-mNG and TUBG1-mRuby2 in the presence of antioxidants (3-min intervals). T=0 is assigned as NEBD. The movie corresponds to Fig. 3a.

File name: Supplementary Movie 4
Description: Mitotic progression in the short-interval live imaging.
Time-lapse imaging of mitotic RPE1 cells expressing H2B-mNG and TUBG1-mRuby2 in the control condition (3-min intervals, left) and short-interval condition (30-sec intervals, center and right). T=0 is assigned as NEBD. The movie corresponds to Fig. 5b.
